# Hydrogen Peroxide Scavenging Activity of Novel Coumarins Synthesized Using Different Approaches

**DOI:** 10.1371/journal.pone.0132175

**Published:** 2015-07-06

**Authors:** Ahmed A. Al-Amiery, Yasameen K. Al-Majedy, Abdul Amir H. Kadhum, Abu Bakar Mohamad

**Affiliations:** 1 Department of Chemical and Process Engineering, University Kebangsaan Malaysia (UKM), Bangi, Selangor, 43000, Malaysia; 2 Environmental Research Center, University of Technology (UOT), Baghdad, 10001, Iraq; 3 Fuel Cell Institute, University Kebangsaan Malaysia (UKM), Bangi, Selangor, 43000, Malaysia; The University of Iowa, UNITED STATES

## Abstract

New derivatives of 7-hydroxy-4-methylcoumarin were synthesized using a chemical method and a microwave-assisted method to compare the feasibility, reaction times, and yields of the product. The newly synthesized coumarins were characterized by different spectroscopic techniques (FT-IR and NMR) and micro-elemental analysis (CHNS). In vitro antioxidant activities of these compounds were evaluated against hydrogen peroxide and were compared with standard natural antioxidant, vitamin C. Our results reveal that these compounds exhibit excellent radical scavenging activities.

## Introduction

**Fig 1:**
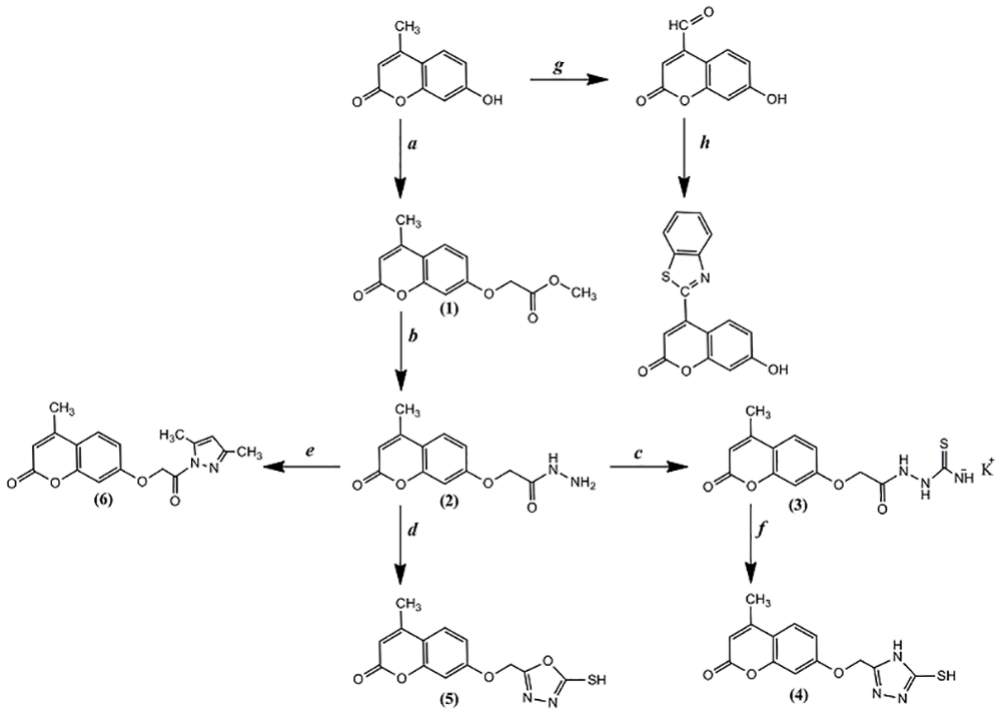
Reaction sequences of the synthesized compounds. *a = Methyl bromoacetate; b = Hydrazine; c = KSCN; d = CS*_*2*_; *e = acetylacetone; f = KOH; g = SeO*_*2*_; *h = o-aminothophenol*.

Coumarins show biological activity, such as molluscicidal, anthelmintic [1], hypnotic and insecticidal [2] activities, as well as medicinal activities, such as anticoagulant agents [3], and as fluorescent brighteners [4]. Coumarins consisting of fused benzene and α-pyrone rings are present in significant amounts in plants, and more than 1300 coumarins have been identified from natural sources [5]. Derivatives of coumarins naturally occur as secondary metabolites present in seeds, roots, and leaves of many plant species [6]. Studies have shown that microwave irradiation substantially aids the promotion and simplification of numerous condensation reactions that can be performed in a solvent and under solvent-free conditions [7–12]. Organic reactions performed using microwave irradiation have rapidly gained popularity because irradiation accelerates the reaction towards a variety of synthetic transformations in solvent-less procedures without using supporting reagents, rendering these reactions eco-friendly [13]. Due to the biological and industrial applications of coumarins and as a continuation of previous studies [14–19], in this study, we describe the synthesis of coumarin derivatives (Fig 1) using chemical and microwave-assisted methods and we describe their characterization through spectral data (FT-IR, ^1^H-NMR) and micro-elemental analysis (CHNS). We also discuss the *in vitro* antioxidant activities of the synthesized coumarins.

## Experimental Section

### General

The chemicals used for synthesis were supplied by Sigma-Aldrich. The IR spectra were obtained on a Thermo (Nicolen 6700) FTIR spectrophotometer, and the values are expressed in cm^-1^. The H-NMR spectra of the compounds were recorded on a Bruker Avance Ii 400 MHz NMR spectrophotometer using DMSO as an internal standard, and the values are expressed in δ ppm. Elemental microanalysis was performed using an Elemental Vario El Iii, Carlo Erba 1108 Elemental analyzer.

#### General procedure for the microwave-assisted synthesis of the compounds

To verify whether microwave irradiation accelerates the final reactions, all of the reactions were performed under microwave irradiation. The reaction time was dramatically reduced for each substitution from 3–12 hr (chemical method) to 1–2 min under microwave irradiation. Microwave-assisted reactions were conducted in septum-sealed reaction vessels in a microwave reactor.

#### Synthesis of ethyl 2-((4-methyl-2-oxo-2H-chromen-7-yl)oxy)acetate (1)

Conventional method: A suspension of 7-hydroxy-4-methylcoumarin (1.086 g, 6.17 mmol) in acetone (30 mL) was refluxed with ethyl bromoacetate (1.528 g, 9.15 mmol) and potassium carbonate K_2_CO_3_ (4.69 g, 33.91 mmol) for 12 hr. After cooling, the mixture was evaporated to dryness, and the residue was partitioned between CHCl_3_ (50 mL) and water (50 mL). The organic phase was dried using Na_2_SO_4_, filtered, and evaporated to dryness. The residue was recrystallized from acetone [20]. Yield 75%; M.p. 98–99°C [lit. [21] 94–96°C]; ^1^H-NMR δ: 2.39 (s, 3H, CH_3_), 3.59 (s, 3H, CH_3_–O), 4.89 (s, 2H, CH_2_); 6.23 (s, 1H, H-3), 6.90 (d, 1H, H-6), 6.93 (s, 1H,H-8), 7.61 (d, 1H, H-5),; IR: 3129 cm^-1^ (C–H, Aromatic), 2924 cm^-1^ (C–H, Aliphatic), 1743.5 cm^-1^ (C = O, ester), 1677 cm^-1^ (C = O, Lacton); Theoretical Calculation for C_13_H_12_O_5_: C 64.12%, H 5.38%. Experimental: C 64.70%, H 5.53%.

#### Synthesis of 2-((4-methyl-2-oxo-2H-chromen-7-yl)oxy)acetohydrazide (2)

Conventional method: A solution of compound **1** (2.48 g, 10 mmol) in 25 mL of ethanol was refluxed with hydrazine hydrate (7.5 g, 15 mmol) for 4 hr. After concentrating the reaction mixture, an oily mass was separated and recrystallized using ethanol [15]. Yield 60%; M.p. 203–205°C [lit. [21] 198–200°C, lit. [22] 202–204]; ^1^H-NMR δ: 2.28 (s, 2H, NH_2_), 4.24 (s, 2H) for (O–CH_2_), 5.86 (s, 1H) for (–C = C–H), 6.38, 6.83, 6.86, 7.45, (s, 1H) for aromatic ring, and 7.50 (s, 1H, NH); IR: 3432.8, 3327.9 cm^-1^ (N–H), 3081.5 cm^-1^ (C–H, Aromatic), 2990.1 cm^-1^ (C–H, Aliphatic), 1670.3 cm^-1^ (C = O, Lactone), 1652.0 cm^-1^ (C = O, Amide); Theoretical Calculation for C_12_H_12_N_2_O_4_: C 58.06%, H 4.87%, N 11.29%. Experimental: C 57.81% H 4.33%, N 10.93%.

Microwave irradiation method: This involves the irradiation of a mixture of Ethyl 2-((4-methyl-2-oxo-2H-chromen-7-yl)oxy)acetate **1**, (2.48 g, 10 mmol) and hydrazine hydrate (7.5 g, 15 mmol) in a microwave oven at 20% intensity for 2 min. After completion of reaction (by TLC), the product was recrystallized from ethanol. The yield of product was 75%. M.p. 199–201°C.

#### Synthesis of potassium (2-(2-((4-methyl-2-oxo-2Hchromen-7-yl)oxy)acetyl)hydrazinecarbonothioyl) amide (3)

Conventional method: A mixture of compound **2** (0.656 g, 2.8 mmol) and KSCN (0.5 g, 5.1 mmol) in 50 mL ethanol was refluxed for 3 hr with a few drops of concentrated HCl [16]. The precipitate formed was collected by filtration and dried to yield compound (**3**). Yield 45%, M.p. over 300°C; ^1^H-NMR δ: 2.33 (s, 3H, CH_3_), 4.23 (s, 2H, CH_2_), 6.12 (s, 1H, H-3), 6.81 (s, 1H, H-8), 6.92 (d, 1H, H-6), 7.70 (d, 1H, H-5), 8.01 (s, 1H, NH), 9.72 (s, 1H, NH), 9.78 (s, 1H, NH); IR: 3346, 3328 cm^-1^ (N–H), 3053 cm^-1^ (C–H, Aromatic), 2937 cm^-1^ (C–H, Aliphatic), 1701 cm^-1^ (C = O, Lactone), 1675 cm^-1^ (C = O, Amide) and 1395 cm^-1^ (C–N); Theoretical Calculation for C_13_H_12_KN_3_O_4_S: C 45.20%, H 3.50%, N 12.17%, S 9.28%. Experimental: C 44.95%, H 3.11%, N 12.00%, S 8.99%.

Microwave irradiation method: This involves the irradiation of compound **2** (0.656 g, 2.8 mmol), KSCN (0.5 g, 5.1 mmol) and few drops of concentrated hydrochloric acid in a microwave oven at 10% intensity for 2 min. After completion of reaction (by TLC), the product was recrystallized from ethanol. The yield of product was 60%. M.p. over 300°C.

#### Synthesis of 7-((5-mercapto-4H-1,2,4-triazol-3-yl)methoxy)4-methyl-2H-chromen-2-one (4)

Conventional method: A mixture of compound **3** (0.431 g, 1.3 mmol), which was used without further purification, and KOH (0.09 g, 1.6 mmol) in 25 mL of water was refluxed for 3 hr [17]. The reaction mixture was cooled and then acidified with HCl to yield compound 4. Yield 35%; M.p. 100–103°C; ^1^H-NMR δ: 2.29 (s, 3H, CH_3_), 3.28 (br s, 1H, -SH), 4.01 (s, 2H, CH_2_), 6.45 (s, H, H-8), 6.73 (d, H, H-6), 7.69 (d, 1H, H-5), 8.33 (s, 1H, NH); IR: 3405, 3252 cm^-1^ (N–H), 3193 cm^-1^ (C–H, Aromatic), 3055 cm^-1^ (C–H, Aliphatic), 2663 cm^-1^ (S–H), 1652 cm^-1^ (C = O, Lactone), 1603 cm^-1^ (C = N), and 1411.4 cm^-1^ (C–N); Theoretical Calculation for C_13_H_11_N_3_O_3_S: C 53.97%, H 3.83%, N 14.52%, S 11.08%. Experimental: C 52.64%, H 3.43%, N 13.89%, S 11.01%.

Microwave irradiation method: This involves the irradiation of compound **3** (0.431 g, 1.3 mmol) and KOH (0.09 g, 1.6 mmol) in a microwave oven at 10% intensity for 1 min. After completion of reaction (by TLC), the product was recrystallized from ethanol. The yield of product was 50%. M.p. 99–101°C.

#### Synthesis of 7-((5-mercapto-1,3,4-oxadiazol-2-yl)methoxy)4-methyl-2H-chromen-2-one (5)

Conventional method: A solution of potassium hydroxide (0.84 g, 15 mmol) in 10 mL of ethanol was added to a mixture of compound **2** (2.48 g, 10 mmol) in 150 mL of ethanol, followed by the addition of carbon disulfide (20 mL). The reaction mixture was heated under reflux for 6 hr and was then concentrated and acidified with dilute HCl. The resulting solid was separated, washed with water, and recrystallized from a mixture of DMF-H_2_O [18, 19]. Yield 60%; M.p. 177–179°C; ^1^H-NMR δ: 2.29 (s, 3H, CH_3_), 3.29 (br s, 1H,-SH), 4.01 (s, 2H, CH_2_), 6.21 (s, 1H, H-3), 6.88 (s, 1H, H-8), 6.77 (d, 1H, H-6), 7.69 (d, 1H, H-5); IR: 3195.1 cm^-1^ (C-H, Aromatic), 3089 cm^-1^ (C-H, Aliphatic), 2765 cm^-1^ (S-H), 1679 cm^-1^ (C = O Lactone) and 1403 cm^-1^ (C-N); Theoretical Calculation for C_13_H_10_N_2_O_4_S: C 53.79%, H 3.47%, N 9.65%, S 11.05%. Experimental: C 53.40%, H 3.11, N 9.31, S 10.97.

Microwave irradiation method: This involves the irradiation of mixture of compound **2** (2.48 g, 10 mmol) and KOH (0.84 g, 15 mmol) in 10 mL of ethanol with carbon disulfide (20 mL) in a microwave oven at 10% intensity for 2 min. After completion of reaction (by TLC), the product was recrystallized from ethanol. The yield of product was 70%. M.p. 180–181°C.

#### Synthesis of 7-(2-(3,5-dimethyl-1H-pyrazol-1-yl)-2-oxoethoxy)4-methyl-2H-chromen-2-one (6)

Conventional method: A mixture of compound **2** (0.248 g, 1 mmol), acetyl acetone (0.142 g, 1 mmol), and acetic acid (1.0 mL) was refluxed in 10 mL of ethanol for 5 hr. The precipitate was collected by filtration and recrystallized from ethanol [19] to yield compound 6. Yield 54%; M.p. 298–299°C; ^1^H-NMR δ: 2.21 (s, 3H, CH_3_), 2.29 (s, 3H, CH_3_), 2.43 (s, 3H, CH_3_), 5.01 (s, 2H, CH_2_), 6.11 (s, 1H, H-3), 6.15 (s, 1H, HC = C), 6.84 (s, 1H, H-8), 6.89(d, 1H, H-6), 7.51 (d, 1H, H-5); IR: 3122 cm^-1^ (C–H, Aromatic), 2985 cm^-1^ (C–H, Aliphatic), 1674.2 cm^-1^ (C = O Lactone), 1395 cm^-1^ (C–N); Theoretical Calculation for C_17_H_16_N_2_O_4_: C 65.38%, H 5.16%, N 8.97%. Experimental: C 65.00%, H 4.93%, N 8.41%.

Microwave irradiation method: This involves the irradiation of mixture of compound **2** (0.248 g, 1 mmol), acetyl acetone (0.142 g, 1 mmol), and acetic acid (1.0 mL) in a microwave oven at 20% intensity for 2 min. After completion of reaction (by TLC), the product was recrystallized from ethanol. The yield of product was 60%. M.p. 297–299°C.

#### Synthesis of 7-hydroxy-2-oxo-2H-chromene-4-carbaldehyde (7)

Conventional method: 7-Hydroxy-4-methylcoumarin (1 g, 0.5 mmole) was dissolved in hot xylene (50 mL). The solution was cooled, and selenium dioxide (1 g, 0.9 mmol) was added. The mixture was refluxed for 12 hr and was filtered while hot. The solvent was removed to obtain the desired product. Yield 55%; ^1^H-NMR δ: 5.28 (s, OH), 6.59 (s, 1H, H-8), 6.61 (d, 1H, H-6), 7.56 (d, 1H, H-5), 7.01 (s, 1H, H-3), 9.59 (s, 1H, CH). IR: 2925.2 cm^-1^ (C–H, Aliphatic), 1743 cm^-1^ (C = O), 1636 cm^-1^ (C = C). Theoretical Calculation for C_10_H_6_O_4_: C 63.16%, H 3.18%, Experimental: C 62.89%, H 3.03%.

Microwave irradiation method: This involves the irradiation of mixture of compound **1** (1 g, 0.5 mmol) and selenium dioxide (1 g, 0.9 mmol) with 50 mL of xylene in a microwave oven at 20% intensity for 2 min. After completion of reaction (by TLC), the product was recrystallized from ethanol. The yield of product was 65%.

#### Synthesis of 4-(benzo[d]thiazol-2-yl)-7-hydroxy-2H-chromen-2-one (8)

Conventional method: Compound **7** (0.95 g, 5.0 mmol) and *o-*aminothiophenol (0.625 g, 5.0 mmol) were refluxed in acetic acid (7 mL) for 5 hr. The solution was cooled, and the product precipitated. This solid was collected, washed with water, and recrystallized from methanol [15]. Yield 75%; M.p. 295–296°C; ^1^H-NMR δ: 5.22 (s, OH), 6.31 (d, 1H, H-6), 6.55 (s, 1H, H-8), 6.78 (s, ^1^H, H-3), 7.34 (t, 1H, *J* = 7.32Hz, H-5), 7.54 (d, 1H, H-5), 7.58 (d, 1H, *J* = 7.57Hz, H-4), 8.21 (t, 1H, *J* = 8.21Hz, H-6), 8.27 (d, 1H, *J* = 8.25Hz, H-7); IR 2921 cm^-1^ (C–H, Aliphatic), 1765.1 cm^-1^ (C = O), 1630 (C = C), 1560.11 cm^-1^ (C = N), 1438 cm^-1^ (C–N), 763.5 cm^-1^ (C–S), Theoretical Calculation for C_10_H_9_NO_3_S: C 65.07%, H 3.07%, N 4.74%, Experimental: C 64.89%, H 3.00%, N 4.32%.

Microwave irradiation method: This involves the irradiation of mixture of compound **7** (0.95 g, 5.0 mmol) and *o-*aminothiophenol (0.625 g, 5.0 mmol) with acetic acid (7 mL) in a microwave oven at 10% intensity for 1 min. After completion of reaction (by TLC), the product was recrystallized from methanol. The yield of product was 80%. M.p. 292–294°C.

### Hydrogen Peroxide Scavenging Activity

A solution of hydrogen peroxide (40 mM) was prepared in phosphate buffer (pH 7.4). Different concentrations (250, 500, and 1000 μg/mL) of the synthesized compounds (or ascorbic acid as the control) were added to a hydrogen peroxide solution (0.6 mL, 40 mM). The absorbance of hydrogen peroxide at 230 nm was determined after 10 min against a blank solution containing phosphate buffer without hydrogen peroxide [22, 23]. The hydrogen peroxide percentage scavenging activity was then calculated using the following equation:

$$H_{2}O_{2\;scavenginig\;effect\;}\%\;=\;A^{{}^{\circ}}-\frac{A}{A^{{}^{\circ}}}\times 100$$ (1)

where *A*_*o*_ is the absorbance of the control reaction and *A*_1_ is the absorbance in the presence of the samples or standards.

### Statistical Analysis

The results were expressed as mean ± standard deviation and the statistical significance of differences were determined utilizing one-way analysis of variance (ANOVA) using the SPSS 17.0 statistical software program. Differences were considered significant at P < 0.05. The values are presented as mean ± SD (n = 3).

## Results and Discussion

### Chemistry

All of the reactions were completed under microwave irradiation and normal reflux conditions, as shown in Table 1. The sequence for the synthesis of the coumarin derivatives 1–10 is shown in Fig 1, starting from 7-hydroxy-4-methylcoumarin. Compound (1), namely, methyl 2-((4-methyl-2-oxo-2H-chromen-7-yl)oxy)acetate, was synthesized by the reflux of methyl bromoacetate, 7-hydroxy-4-methylcoumarin, anhydrous potassium carbonate and anhydrous acetone. The FT-IR spectrum for compound 1 showed an absorption band at 1743.5 cm^-1^ due to the stretching of the esteric carbonyl (-C = O). The ^1^H-NMR spectrum showed a singlet at δ 3.65 ppm due to the methyl protons (3H of CH_3_) and a singlet at δ 3.68 ppm due to methylene protons (2H of CH_2_). Compound 1 was reacted with hydrazine hydrate to afford hydrazide 2 in good yield. Compound 2 (hydrazide) showed absorption bands at 3231.5, 3225.1, and 3209 cm^-1^ (hydrazide NH-NH_2_). The ^1^H-NMR spectrum exhibited a singlet due to the (s, 2H, CH_2_) proton at δ 4.24 ppm and a singlet due to the (1H, NH) proton at δ 7.50 ppm. Compound 2 was refluxed with KSCN in ethanol as the solvent containing catalytic amounts of HCl to yield salt 3, which was converted directly to 4 in good yield by heating in aqueous KOH followed by acidification with HCl. Compound 5 was prepared accordingly by heating 2 with CS_2_ in the presence of ethanolic potassium hydroxide. By condensation of 2 with acetyl acetone in ethanol with a few drops of acetic acid, the corresponding derivative 6 was obtained in 54% yield. Upon condensation of 2 with xylene and selenium dioxide, the corresponding derivative 7 was obtained in good yield. Compound 8 was also obtained by refluxing compound 7 with *o*-aminothiophenol.

**Table 1:** Comparison between the microwave-assisted and chemical methods of synthesis in terms of yield and time.

Compound	Microwave Method		Chemical Method	
	Time (min)	Yield (%)	Time (hr.)	Yield (%)
1	-	-	12	75
2	2	75	4	60
3	2	60	3	45
4	1	50	3	35
5	2	70	3	60
6	2	60	5	54
7	2	65	12	55
8	1	80	5	75

#### Scavenging Activity

**Fig 2:**
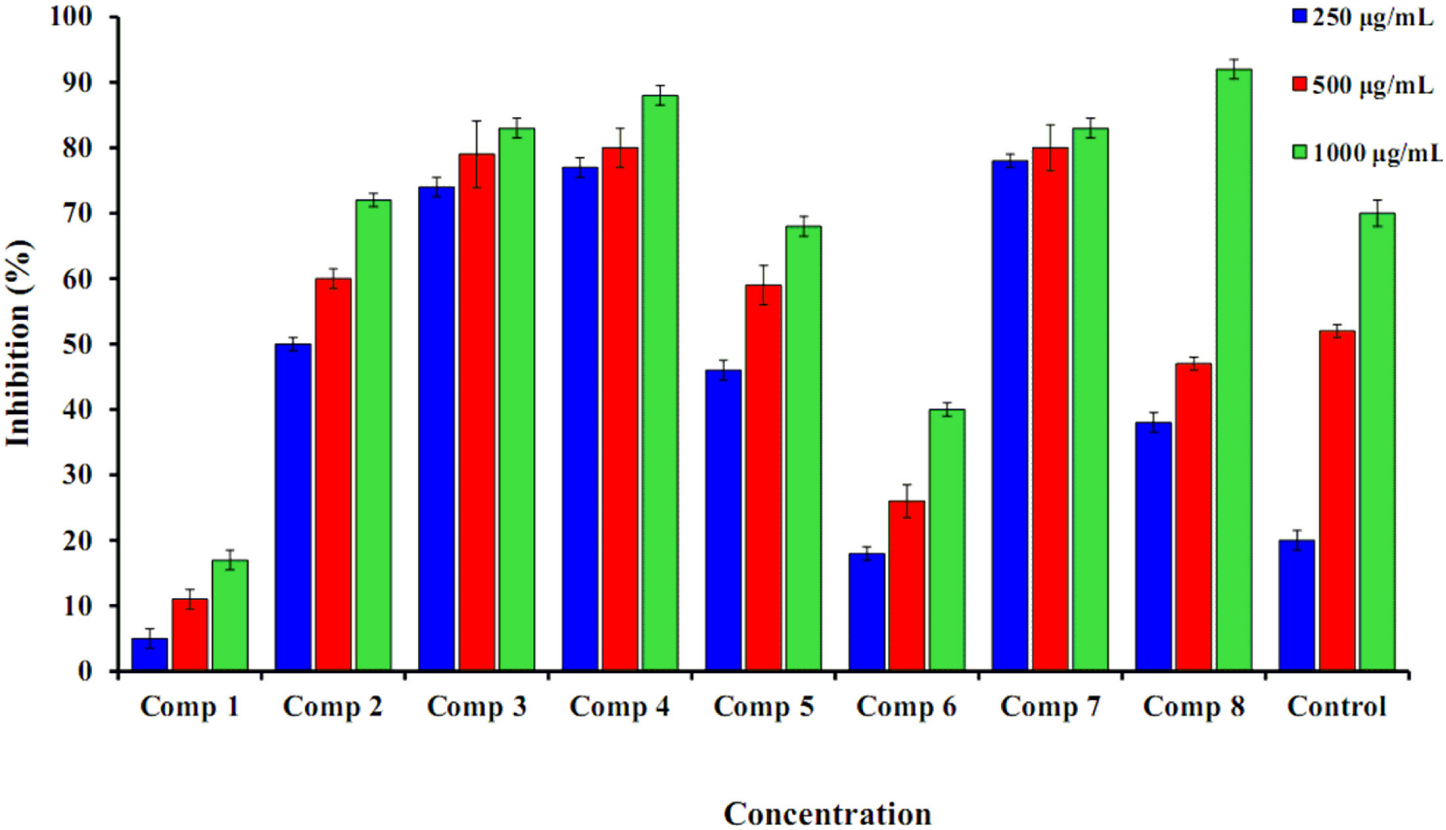
Percentage inhibition of hydrogen peroxide scavenging activity of synthesized compounds (1–8) in comparison to Vitamin C. n = 3. Error bars indicate standard deviation.

The role of a cancer prevention agent is to remove free radicals. The most important mechanism to achieve this goal is the donation of hydrogen to free radicals to convert them to nonreactive species [24]. The donation of hydrogen would remove the odd electron that is responsible for radical reactivity [25]. Free radicals have been a subject of critical interest among researchers in the previous decade. The wide range of free radical effects in biological systems has garnered interest from many specialists. It has been demonstrated that free radicals assume an important role in the pathogenesis of specific diseases and aging [24,25]. Numerous synthetic cancer prevention agents have presented toxic and/or mutagenic effects; thus, naturally occurring antioxidants have been considered [26]. Synthesized coumarins 1–8 were screened for *in vitro* scavenging activity utilizing hydrogen peroxide. These tested coumarins showed high scavenging activity (Fig 2).

**Fig 3:**
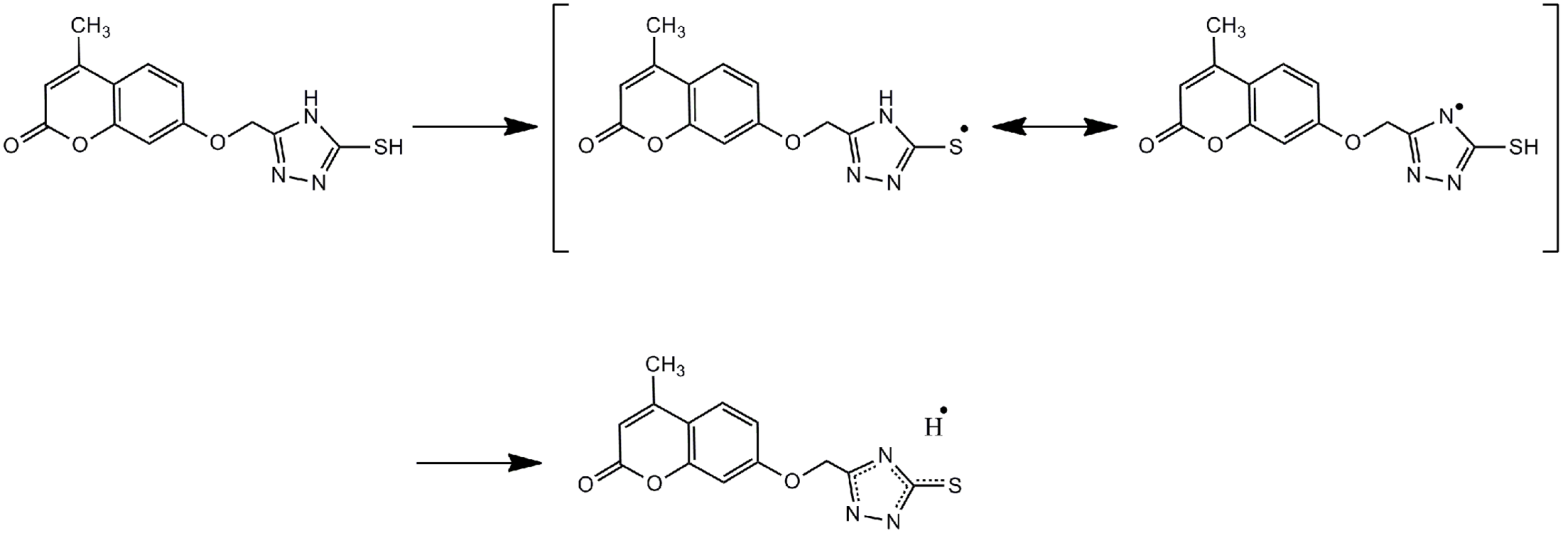
Suggested mechanism for compound 4 as an antioxidant.

Fig 2 showed that the eight synthesized compounds (1–8) demonstrated a strong scavenging activity against H_2_O_2_. At a very low concentration of 250 μg/mL we observed a concentration dependent decrease in H2O2 activity. A very weak inhibitory activity was found in compound 1 and compound 6 (5.33 ± 0.88 and 18.0 ± 1.00). The highest concentration was found at 1000 μg/mL (Fig 2). The best percentage scavenging activity was shown by compound 8 (91.66 ± 1.52), followed by compound 4, 3 and 7 (88.33 ± 1.50; (83.33 ± 1.52 and (82.66 ± 1.52;). However, their activity was not significantly different at 95% confidence interval. vitamin C was used as standard drugs with percentage inhibition of 70.00 ± 2.00. The hydrogen-donating activity, measured utilizing hydrogen peroxide radicals as the hydrogen acceptor, demonstrated that a strong association could be found between the concentration of the coumarin molecule and the rate of inhibition [27]. Using the hydrogen peroxide test, coumarins 1–8 demonstrated their ability to diminish the stable radical. The postulated mechanism for the reaction of coumarin 4 as an antioxidant, as indicated in Fig 3, relies on the mercapto hydrogen atom (bold), which is under the influence of resonance and inductive effects. The resonance effect of the mercapto hydrogen facilitates the release of hydrogen, while the inductive effect pushes the electrons toward a sulfur-free radical, resulting in stability of the molecule.

**Fig 4:**
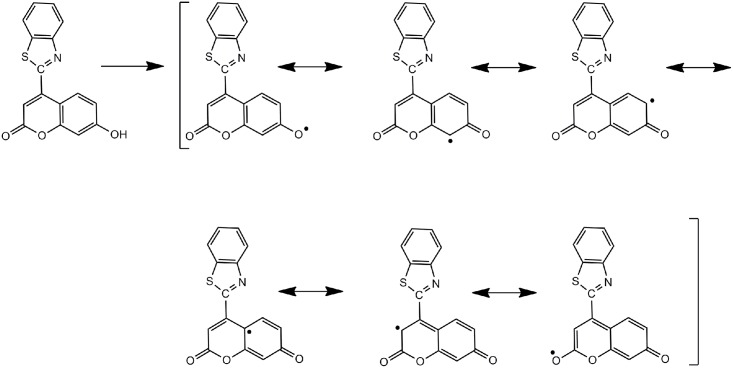
Suggested mechanism for compound 8 as an antioxidant.

The postulated antioxidant mechanism for synthesized coumarin 8, as shown in Fig 4, relies on the hydroxyl hydrogen atom (bold), which is under the influence of resonance and inductive effects. The resonance effect of the oxygen hydrogen facilitates the release of hydrogen, while the inductive effect pushes the electrons toward the oxygen-free radical, resulting in stability of the molecule [28]. Note that coumarin 8 has a higher scavenging activity because of the stability of the free radical intermediates of this coumarin [29].

## Conclusions

New coumarin derivatives were successfully synthesized using chemical and microwave irradiation methods. The characterized of these coumarins (S1 and S2 Figs) were done by using different spectroscopic techniques (FT-IR and NMR) and micro-elemental analysis (CHNS). The scavenging activity of these coumarins were determined by using hydrogen peroxide assay. Results indicated that the new coumarins possess higher scavenging activity than vitamin C. The availability of these coumarins would also facilitate further investigations of their pharmacological properties.

## Supporting Information

S1 Fig FT-IR spectrum. (PDF)

S2 Fig Proton NMR spectrum. (PDF)
