# Supplementary figures and images for: Hydrogen Peroxide Scavenging Activity of Novel Coumarins Synthesized Using Different Approaches
Source: PLoS One. 2015 Jul 6;10(7):e0132175. doi: 10.1371/journal.pone.0132175 (PMC4492988; doi:10.1371/journal.pone.0132175)

## FT-IR spectrum for compound 2

g 15 14:09:33 2013

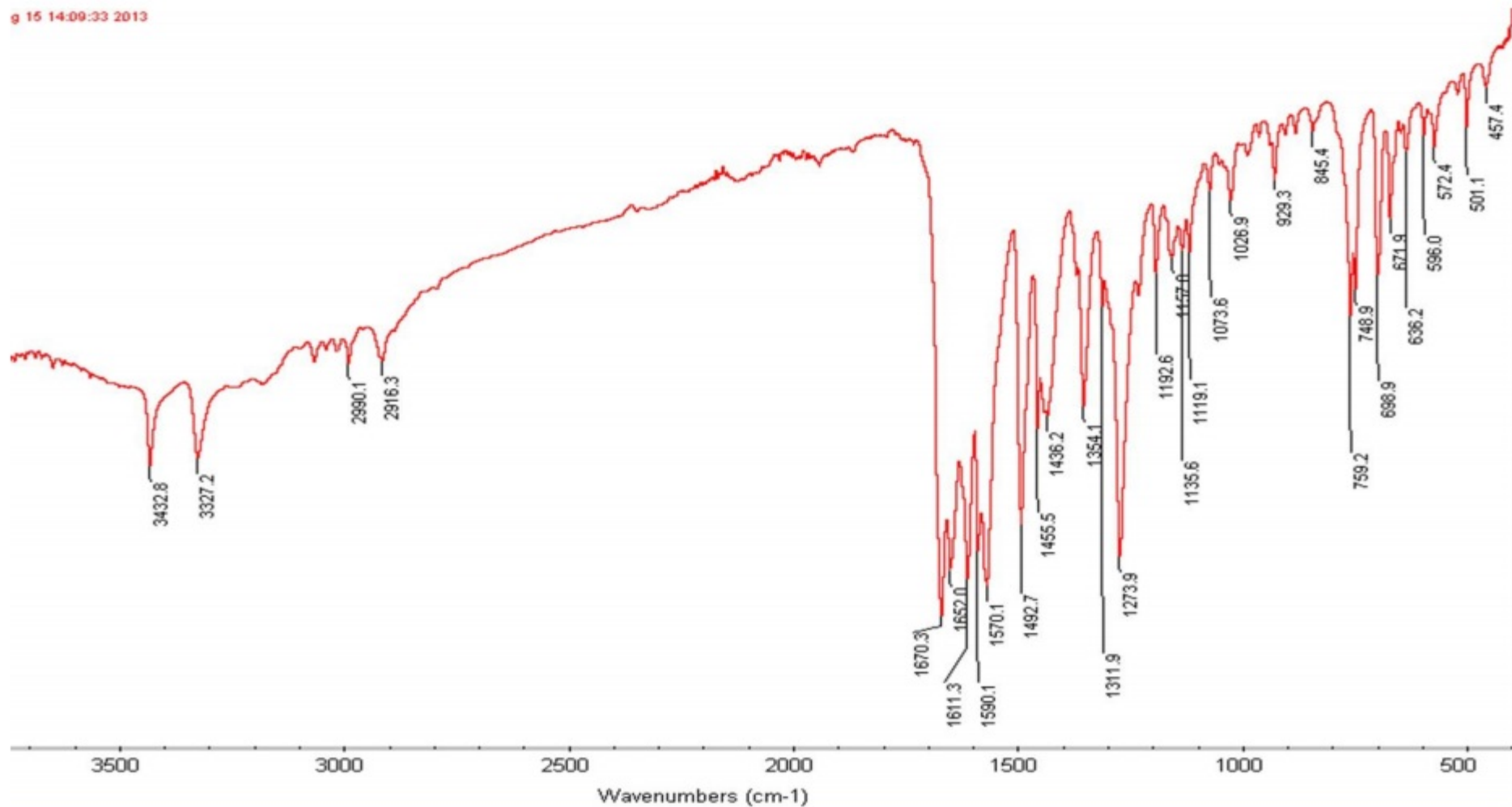

Supplement: S1 Fig — (PDF) [file pone.0132175.s001.pdf]
