# Supplementary material for: Hydrogen Peroxide Scavenging Activity of Novel Coumarins Synthesized Using Different Approaches
Source: PLoS One. 2015 Jul 6;10(7):e0132175. doi: 10.1371/journal.pone.0132175 (PMC4492988; doi:10.1371/journal.pone.0132175)

NMR spectrum for compound 2

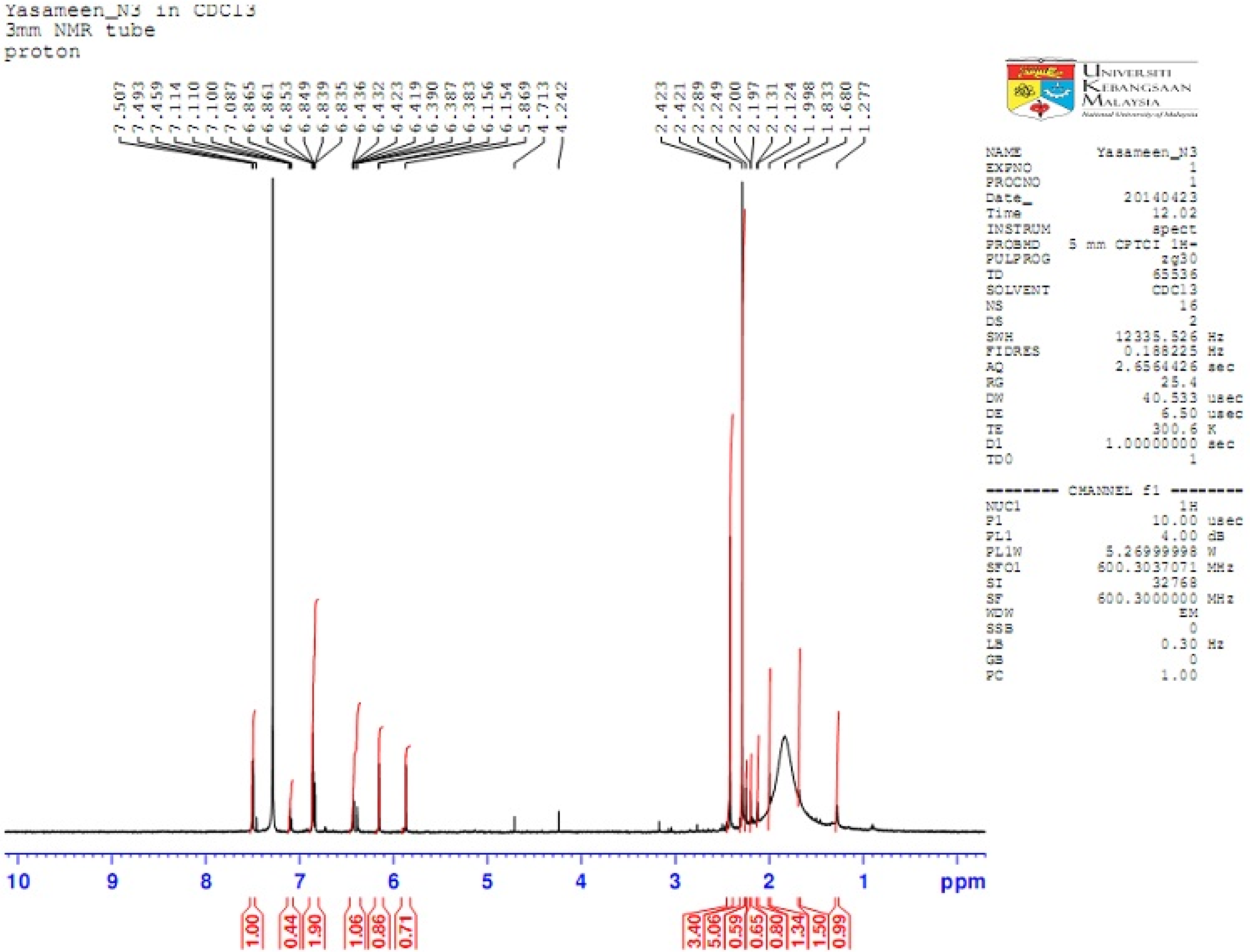

Supplement: S2 Fig — (PDF) [file pone.0132175.s002.pdf]
